# Anti-Inflammatory and Antioxidant Effects of Leaves and Sheath from Bamboo (*Phyllostacys edulis* J. Houz)

**DOI:** 10.3390/antiox12061239

**Published:** 2023-06-08

**Authors:** Rosa Tundis, Giuseppina Augimeri, Adele Vivacqua, Rosa Romeo, Vincenzo Sicari, Daniela Bonofiglio, Monica Rosa Loizzo

**Affiliations:** 1 Department of Pharmacy, Health and Nutritional Sciences, University of Calabria, 87036 Rende, CS, Italy; rosa.tundis@unical.it (R.T.); daniela.bonofiglio@unical.it (D.B.); monica_rosa.loizzo@unical.it (M.R.L.); 2 Department of Agriculture, Mediterranea University of Reggio Calabria, 89122 Reggio Calabria, RC, Italy; rosa.romeo@unirc.it (R.R.); vincenzo.sicari@unirc.it (V.S.)

**Keywords:** bamboo, by-products, leaves, sheath, antioxidant, anti-inflammatory, bioactivity, human cell lines

## Abstract

Bamboo (*Phyllostacys edulis* J. Houz) has become an emerging forest resource of economic and ecological significance with health benefits. Since the beneficial effects of the non-edible parts of bamboo have not been thoroughly explored, we characterized in this study bamboo leaf (BL) and sheath (BS) extracts. The total phenol and flavonoid content (TPC and TFC), antioxidant activity (ABTS, DPPH, FRAP and β-carotene bleaching test) and anti-inflammatory properties were determined. Leaves exhibited a TPC value of 73.92 mg equivalent (eq) gallic acid/g fresh weight (FW) and a TFC value of 56.75 mg eq quercetin/g FW. Ultra-High-Performance Liquid Chromatography (UHPLC) coupled with photo diode array detector (PDA) analysis revealed evidence for the presence of protocatechuic acid, isoorientin, orientin and isovitexin in BL, whereas BS was rich in phenolic acids. Both samples demonstrated a significant ability to scavenge radicals against ABTS^·+^, with an inhibitory concentration of 50% of 3.07 μg/mL for BL and 6.78 μg/mL for BS. At a concentration of 0.1 and 0.2 mg/mL, BS decreased reactive oxygen species production without hampering cell viability in HepG2 liver cells, while at the same concentrations, BL exhibited cytotoxicity in HepG2 cells. In addition, 0.1 and 0.2 mg/mL BS and BL reduced Interleukin-6 and Monocyte Chemoattractant Protein-1 production in human lipopolysaccharide-stimulated THP-1 macrophages, without affecting cell viability. These findings highlight the anti-inflammatory and antioxidant properties of BL and BS, corroborating their different potential applications in the nutraceutical, cosmetic and pharmaceutical industries.

## 1. Introduction

Bamboo (*Phyllostacys edulis* J. Houz) is an economically important member of the family Poaceae, subfamily Bambusoideae. Bamboo species can adapt to a wide variety of climatic conditions and ecosystems. A total of 1718 different bamboo species are currently known and described, divided into 91 genera [1]. However, bamboo cannot be found on all continents. Bamboo resources are especially rich in Asia, where China and India represent the largest bamboo reserve [2]. Among its uses, we find food (shoots), a substitute for wood (the cane, used for support sticks for horticultural crops, furniture, toothpicks, etc.), fuel and, finally, an anti-erosion crop [3,4].

The sheath represents 1/3 of the biomass of the raw bamboo shoot and it is often considered a waste in bamboo shoot industrial processing [5]. Few studies have investigated the phytochemical composition and bioactivity of the sheath. Phenols, flavonoids, dietary fibers, xylan and polysaccharides were identified as its main compounds [6,7,8,9,10]. However, up to now, the limited knowledge surrounding bamboo sheath has strongly influenced how it is used.

Industrial waste re-use can help to achieve a higher level of sustainability [11]. Recently, the European Commission has undertaken a series of actions aimed at minimizing waste through recovery and regeneration methods which re-introduce waste, even in a different form, into a new life cycle. In this context, significant attention has been given to the recovery of healthy molecules from various by-products, which may be useful for the development of functional foods or nutraceutical products [12].

In recent decades, research interest in natural plant antioxidant and anti-inflammatory activities has been promising and innovative with respect to potential applications in fields such as food, cosmetics and pharmaceuticals [13,14,15]. When endogenous and exogenous antioxidants fail to counteract free radicals, a condition of oxidative stress is generated, resulting in cellular damage and related diseases [16]. Many natural compounds rich in bioactive molecules, including bamboo, are potential antioxidants, able to protect against reactive oxygen species (ROS) and, ultimately, ameliorate oxidative stress–related diseases, such as cancer, cardiovascular and neurodegenerative diseases and inflammatory disorders [17,18,19].

In addition, the bioactive agents naturally contained in plants are important in defence responses due to their anti-inflammatory properties, proving to be beneficial for human health [20]. Mounting evidence has shown a close link between inflammation and a wide range of chronic health conditions, although the mechanism by which inflammation is involved in pathological alterations is still not fully understood. Nevertheless, the state of chronic inflammation usually involves an imbalance of pro-inflammatory cytokines and anti-inflammatory cytokines. Several studies have proven that extracts and their constituents exhibit anti-inflammatory properties by blocking signaling pathways which play a key role in the production of pro-inflammatory mediators, or by inhibiting pro-inflammatory cytokines [21].

The anti-inflammatory activities of bamboo leaf extracts specifically have been described in different cellular contexts [22,23,24]. Despite the growing identification of chemical compounds in bamboo, most of the pharmacological studies have focused on bamboo leaf flavonoids [19]. Thus, further studies are needed to evaluate the activities of different bamboo by-products and to perform toxicity studies on this plant. 

In this context, this work aimed to evaluate the potential bioactivity of two processing by-products of bamboo: leaves and sheaths. For this purpose, the phytochemical profile of bamboo extracts was studied, along with their potential antioxidant and anti-inflammatory activities, using as cellular model systems the human hepatocellular carcinoma HepG2 and THP-1 monocytic cell lines. 

## 2. Materials and Methods

### 2.1. Chemicals and Reagents 

All reagents were obtained from Sigma Aldrich (Milan, Italy), with the following exceptions: solvents, protocatechuic acid, isoorientin, orientin, isovitexin, chlorogenic acid and rutin were purchased from VWR International s.r.l. (Milan, Italy); *p*-Coumaric acid was purchased from Fluka (Steinheim, Germany); caffeic acid was procured from Extrasynthèse (Lyon, France).

### 2.2. Extraction Process 

The leaves (the size of about 7–10 × 40 cm) and shoots of bamboo (*Phyllostacys edulis*) were collected in April 2022 in Falco Farm (Corigliano Calabro, Cosenza, Italy) (39°35′45″60 N; 16°31′6″60 E; 210 m above sea level). Leaves were washed and cut into small pieces for subsequent extraction. Shoots were manually peeled to remove the sheath for further analysis. Both leaves and shoots (500 g) were subjected to ultrasound-assisted maceration using a hydroalcoholic solution of ethanol/water (8:2 *v*/*v*, 700 mL) as solvent in a Branson model 3800-CPXH water bath (Branson, Milan, Italy) with a frequency of 40 kHz at 25 °C for 45 min. The extraction procedure was repeated 4 times and after each extraction cycle, solutions were filtered and the solvent was removed using a rotary vacuum evaporator. 

### 2.3. Phytochemical Content

The determination of total phenol content (TPC) was carried out following the procedure previously reported [25]. For total flavonoid content (TFC) evaluation, the procedure described by Leporini et al. [25] was carried out. Results were reported as mg of chlorogenic acid equivalents (CAE)/g of fresh weight (FW) for TPC and mg quercetin equivalents (QE)/g of FW for TFC. 

### 2.4. UHPLC Quantification of Mainly Phenolic and Flavoinoid Compounds

Ultra-High-Performance Liquid Chromatography (UHPLC) PLATINblue (Knauer, Berlin, Germany) with a PDA-1 (photo diode array detector) was used for quantification of mainly phenolic and flavoinoid compounds occurring in bamboo leaves and sheaths [26]. For chromatographic separation, a C18 column (Knauer, 1.8 µm, 150 *×* 3 mm) with a mobile phase consisting of H_2_O (pH 3.10 with acetic acid) (A) and CH_3_CN (B) was used. Compounds were detected by UV absorption at 245 nm, 280 nm and 303 nm. The gradient elution is reported in Table S1 (Supplementary Materials). External standards were used for the quantification of each individual component. 

### 2.5. In Vitro Antioxidant Activity 

To evaluate the ability of bamboo extracts to counteract oxidative stress, various in vitro methods, based on different mechanisms of action, were assessed. For the evaluation of radical scavenging activity, 2,2-diphenyl-1-picrylhydrazyl (DPPH) and 2,2-azino-bis(3-ethylbenzothiazoline-6-sulfonic) acid (ABTS) assays were used [26]. For the ABTS test, ABTS radical solution was combined with bamboo samples at different concentrations, and after incubation the absorbance was read at 734 nm [25]. In the DPPH assay, the radical was prepared and mixed with bamboo extracts. After 30 min of incubation, the bleaching of the radical was measured at 517 nm. In both tests, ascorbic acid was used as a positive control. 

The ability of extracts to induce the reduction of tripyridyltriazine (TPTZ)-Fe^3+^ was evaluated spectrophotometrically at 595 nm, using a Ferric reducing ability of plasma (FRAP) assay, whereas a β-carotene bleaching test was used to determine the inhibition of the lipid peroxidization in a liposome model system [26]. Butylhydroxytoluene (BHT) and propyl gallate were used as the positive controls in the FRAP and β-carotene bleaching test, respectively.

### 2.6. Cell Culture 

HepG2 and THP-1 cell lines (American Type Culture Collection, ATCC, Manassas, VA, USA) were authenticated and stored in accordance with the instructions provided by the supplier. HepG2 and THP-1 cells were cultured in Eagle’s Minimum Essential Medium (ATCC) and Roswell Park Memorial Institute (RPMI)-1640 medium (Lonza, Verviers, Belgium), respectively, in the presence of 10% fetal bovine serum (FBS, Life Technologies, Monza, Italy) and 1% penicillin-streptomycin in a humidified 5% CO_2_ atmosphere at 37 °C. THP-1 monocytes were plated and differentiated into macrophages (M0) with phorbol 12-myristate 12-acetatate (PMA) 100 nM for 24 h and then within a medium without PMA for 1 day, as described by Gionfriddo et al. [27]. M0 macrophages were exposed to 0.1 and 0.2 mg/mL of BL and BS, dissolved in distilled water and dimethylsulfoxide (DMSO), respectively, prior to treatment for 24 h with Lipopolysaccharide (LPS) 10 ng/mL, which was able to polarize M0 into the inflammatory M1 macrophages. 

### 2.7. Cell Viability Assay

HepG2 and THP-1 cells were plated and treated with 0.1 and 0.2 mg/mL of BL and BS for 24 h. A 3-(4,5-dimethylthiazol-2-yl)-2,5-diphenyltetrazolium (MTT) assay was used to evaluate cell viability, as previously reported [28]. The absorbance was measured in Multiskan SkyHigh Photometer at a test wavelength of 570 nm (Thermo Fisher Scientific, Milan, Italy). Viable cells were expressed as a percentage with respect to the control (100%). 

### 2.8. Real-Time PCR Assays

Extraction of total RNA was performed as previously described [29]. Real-time PCR was used to analyze cDNA in an iCycler iQ Detection System (Bio-Rad, Hercules, CA, USA). cDNA was determined in duplicates using SYBR Green Universal PCR Master Mix. Each sample was normalized by 18S mRNA content, as previously reported [30]. For gene amplification Interleukin 6 (IL-6, Gene ID: 3569): forward 5′-CCAGGAGCCCAGCTATGAAC-3′ and reverse 5′-CCCAGGGAGAAGGCAACTG-3′; Monocyte Chemoattractant Protein-1 (MCP-1, Gene ID: 6347): forward 5′-CAGCCAGATGCAATCAATGCC-3′ and reverse 5′-TGGAATCCTGAACCCACTTCT-3′; 18s rRNA (18S, Gene ID: 106631781): forward 5′-CCCACTCCTCCACCTTTGAC-3′ and reverse 5′-TGTTGCTGTAGCCAAATTCGTT-3′ primers were used.

### 2.9. Measurement of Intracellular Reactive Oxygen Species Production

Cells were exposed to BL and BS at concentrations of 0.1 and 0.2 mg/mL for 4 h, prior to treatment with 10 mM of hydrogen peroxide (H_2_O_2_) for 30 min. Cells treated with H_2_O_2_ were used as positive controls. Then, CellROX^®^ Green Reagent (5 μM), a fluorogenic probe designed to reliably measure the levels of intracellular reactive oxygen species (ROS) in live cells, was added to the medium for 30 min at 37 °C. Upon oxidation, it binds to DNA, localizing mainly in the nucleus and mitochondria. After incubation, cells were washed with PBS and fixed for 15 min with 3.7% formaldehyde. Intracellular ROS levels were evaluated by a Olympus BX51 fluorescence microscope (Olympus, Tokyo, Japan) 10× objective and quantified by ImageJ. 

### 2.10. Statistical Analysis

Data are expressed as means ± standard deviations (S.D.) or standard error mean (SEM). Prism GraphPad Prism Software (GraphPad Software, San Diego, CA, USA) was used to calculate the concentration giving 50% inhibition (IC_50_). In the total phytochemical content, Tukey’s test was used to determine any significant difference among investigated samples. In the antioxidant assays, differences within and between groups were evaluated by ANOVA followed by the Dunnett’s test. In cell culture-based tests, differences between means were analyzed by the Student’s t-test. Pearson’s correlation coefficient, mean, SD or SEM calculation were completed using Microsoft Excel (Microsoft, Redmond, WA, USA). 

## 3. Results and discussion

### 3.1. Extraction Yield, Phytochemical Contents and UHPLC Analysis

Extraction yields of 12.45 and 6.43% were obtained with leaves and sheath, respectively. The TPC ranged from 19.36 to 73.92 mg eq CAE/g FW for BL and BS samples, respectively, whereas values from 4.69 to 56.75 mg eq QE/g FW were recorded for TFC. 

The impact of the extraction procedure on the TPC and TFC of the leaves of three commonly cultivated bamboo species (*Gigantochloa verticillate*, *G. atter* and *Dendrocalamus asper*) collected in Indonesia were previously investigated. Ethyl acetate extracts were richest in TPC with values in the range of 25.50–26.09 mg GAE/g for *G. verticillata* and *D. asper*, respectively, followed by ethanol 70% (*v*/*v*) and hot water extracts. A similar situation was observed for TFC with values in the range of 84.03 to 92.67 mg QE/g for *G. atter* and *G. verticillate*, respectively [31]. All these values are in agreement with our results. 

More recently, Benjamin et al. [32] evaluated the impact of drying methods (freeze-drying, microwave, oven, shade and sun) on the leaves of six different species of bamboo from Malaysia, namely *Dinochloa sublaevigata*, *Bambusa tuldoides*, *B. vulgaris*, *B. multiplex*, *Schizostachyum brachycladum* and *Gigantochloa levis.* Results clearly showed that the drying process considerably affects the retention of phytochemicals in the plant matrix. Leaves subjected to the freeze-drying process are characterized by the highest TPC and TFC, with values from 2.69 to 12.59 mg GAE/g for *G. levis* and *D. sublaevigata*, and 0.87–2.12 mg QE/g for *G. levis* and *S. brachycladum*, respectively. Values of 14.6 mg GAE/g powder and 6.71 mg quercetin equivalent/g powder were found for *Bambusa arundinacea* methanol extracts [33]. TPC values in the range of 28.056–29.586 mg GAE/g for *Phyllostachys Tao Kiang* and *P. pubescens* leaves ethanol (80%) extract obtained by an ultrasound-assisted maceration process, and TFC values in the range of 17.678–25559 mg rutin eq/g for *Phyllostachys aureosuleata* and *P. heterocycla*, respectively, were found [8]. 

Compared to the leaves, much less investigated is the plant matrix derived from the shoot processing, namely the external sheath. Previously, Jiang et al. [5] investigated the TPC in *Phyllostachys pracecox* sheath and found a high TPC (85.3 mg GAE/g DW), whereas values in the range of 9.103–16.692 mg GAE/g for *Phyllostachys spectabilis* and *P. heterocycle*, respectively, were found by Li et al. [8]. In the same study, TFC levels were quantified in the range of 1.292–6.325 mg rutin eq/g for *P. spectabilis* and *P. heterocycle*, respectively.

Selected phytochemicals were identified in bamboo leaf and sheath extracts (Figure S1). According to Ma et al., 2020 [34], a BL sample chemical profile was characterized by a high concentration of protocatechuic acid (911.14 μg/g), isoorientin (1197.64 μg/g), orientin (302.54 μg/g) and isovitexin (575.1 μg/g), whereas BS was mainly characterized by phenolic acids, particularly protocatechuic acid (650.73 μg/g), chlorogenic acid (742.81 μg/g), caffeic acid (155.57 μg/g) and *p*-cumaric acid (41.20 μg/g). Rutin (119.79 μg/g) was also identified.

Data obtained in our study are in agreement with data found by Wang et al. [35], except with respect to *p*-cumaric acid (41.20 μg/g) and ferulic acid (64.46 μg/g). 

### 3.2. In Vitro Antioxidant Activity

**Figure 1:**
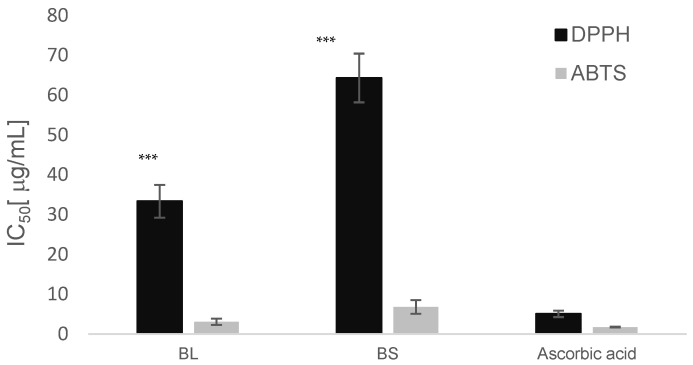
Radical scavenging activity assessed by DPPH and ABTS assays. The values represent the means ± S.D. (*n* = 3). BL: Bamboo leaves; BS: Bamboo sheath. Differences within and between groups were evaluated by One-way ANOVA followed by a multicomparison Dunnett’s test compared to ascorbic acid (*** *p* < 0.001).

Leaf extract showed the most promising radical scavenging potential with IC_50_ of 3.07 and 44.32 μg/mL in the ABTS and DPPH tests, respectively (Figure 1). A promising ABTS radical scavenging activity was also observed for the sheath extract (IC_50_ of 6.78 μg/mL).

The protection of β-carotene from the oxidant activity carried out by the thermo-degradation products of linoleic acid was assessed using a β-carotene bleaching test. The obtained data showed that leaves increase the activity after 60 min of incubation (IC_50_ of 41.04 and 19.61 μg/mL at 30 and 60 min, respectively) (Table 1). A low ferric-reducing ability was found in both samples with FRAP values of 7.71 and 10.25 for BS and BL, respectively (Table 1). A positive correlation was independently found between antioxidant activity and total phytochemical content (TPC and TFC), using the test applied to investigate the bioactivity. 

Several studies have demonstrated the antioxidant potential of the bamboo leaves. The variability of antioxidant activity, in accordance with the analyses in the literature, is related to different factors including bamboo species, place of collection, season of collection, pre-treatment of the plant matrix, solvent used for extraction and extraction procedure.

Ni et al. [36,37] reported the radical scavenging potential and ferric-reducing ability of *Indocalamus latifolius* and *Sasa argenteastriatus*, two bamboo species particularly common in East Asia. Results indicated that the higher altitude reduced the secondary metabolite accumulation and consequently the antioxidant potential. Moreover, the bamboo leaf extract’s DPPH radical scavenging activity was influenced by season, since it was higher in autumn and winter compared to other seasons, with maximum values in the range of 284.08–457.42 μg/mL. Values in the range of 234.57 to 422.87 μmol/L were found using a FRAP test. A similar trend was observed for bamboo leaves from *Phyllostachys* species [38].

Gong et al. [39] investigated the DPPH radical scavenging potential of *Phyllostachys nigra* var. *henonis* leaves and found an IC_50_ value of 1.81 μg/mL, which is significantly lower than those found in our sample. A lower DPPH radical scavenging potential was independently observed for *Gigantochloa atter*, *Dendrocalamus asper* and *Gigantochloa verticillate*, in comparison with our data, using the solvent used for extraction with values in the range of 566.79 to 1825.07 μg/mL for *G. verticillata* and *G. atter*, respectively [31]. An IC_50_ value of 164.11 μg/mL was found with *Bambusa vulgaris* leaf extract [40]. 

A lower DPPH radical scavenging potential was found for three different bamboo species collected in China with IC_50_ values in the range of 2.10–10.17, 1.25–5.07 and 1.59–2.72 mg/mL for *Lophatherum gracile*, *Pleioblastus amarus* and *Phyllostachys nigra*, respectively [34]. A similar situation was also observed when comparing our data with those obtained from the leaves of *Bambusa arundinacea* with IC_50_ values in the range of 273–1103 μg/mL [33].

Data found using the ABTS test are in line with those obtained by Benjamin et al. [32], which found IC_50_ values in the range of 1.89 to 3.47 μg/mL for *B. tuldoides* leaves subjected to oven drying and *S. brachycladum* leaves subjected to sun drying, respectively, whereas all extracts exhibited a higher DPPH radical scavenging potential with IC_50_ values of 2.92–4.73 μg/mL. A comparable radical scavenging potential was observed with data obtained by Kim et al. [41], with extracts obtained using *Phyllostachys nigra* leaves with IC_50_ values in the range of 1.79–32.64 and 2.05–47.90 mg/mL for ABTS and DPPH, respectively.

Li et al. [8] evaluated the DPPH radical scavenging potential and ferric-reducing ability of eight different *Phyllostachys* species and found that the leaves of *P. pubescens* resulted the most active, whereas among the sheath the strongest antioxidant potential was observed in the golden thread moso bamboo. More recently, Cao et al. [42] investigated the impact of the extraction procedure on the recovery of bioactive compounds using *Bambusa chungii* culms processing waste, as well as its influence on antioxidant activity. The best extraction conditions were found at a temperature of 160 °C and with a solvent ratio of 1:30 g/mL for 14 min. The obtained extract showed IC_50_ values of 27.22 and 125.07 mg/L for the ABTS and DPPH tests, respectively. 

A certain variability was observed using the FRAP test with values in the range of 6.40–36.65 mg Trolox equivalent/g. An ABTS radical scavenging potential from 1.373 and 1.650 mg eq di Vitamin C/mL was found for *Phyllostachys pubescens* leaves subjected to different extraction procedures in terms of temperature and time [43].

Regarding sheath, Jiang et al. [5] found values of 33 and 77 μmol ascorbic acid equivalent (AAE)/g DW for FRAP and DPPH, respectively, using *Phyllostachys pracecox* ultrasound-assisted ethanol extract. 

### 3.3. Antioxidant Power of Bamboo Leaves and Sheaths in Human Liver HepG2 Cells

**Figure 2:**
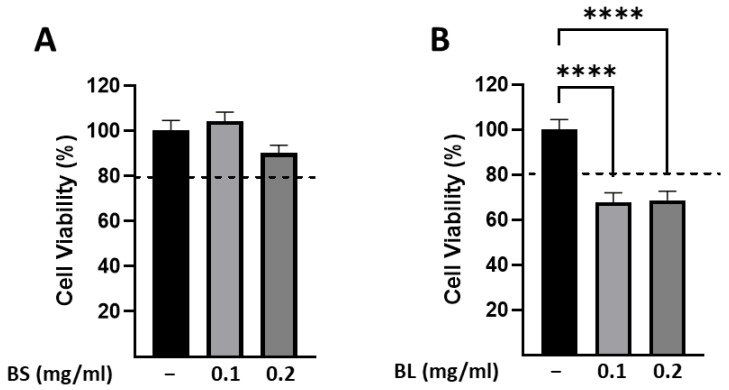
Cell viability assessed by MTT assay in HepG2 cells. HepG2 cells were untreated (−) or treated with 0.1 and 0.2 mg/mL of bamboo sheaths (BS) (**A**) and bamboo leaves (BL) (**B**) for 24 h. The histograms represent the means ± SEM of three independent experiments, each performed in triplicate. **** *p* < 0.0001.

The human hepatoma HepG2 cells are the most commonly used cell model in hepatotoxicity studies since they display an epithelial-like morphology [44]. Based on our results, showing that different in vitro tests revealed the antioxidant activities of extracts derived from bamboo in a dose-dependent manner, we first assessed whether the highest IC_50_ values of both extracts are cytotoxic in HepG2 cells. As shown in Figure 2A, the exposure of cells to BS for 24 h, at concentrations of 0.1 and 0.2 mg/mL, did not result in cell toxicity, while both concentrations significantly reduced cell viability upon treatment with BL (Figure 2B). We hypothesize that the different qualitative and quantitative compositions of the bamboo extracts may influence viability in a cell context-dependent manner. In particular, by chromatography we identified isoorientin in BL, but not in BS extracts, which is an antiproliferative and apoptotic agent in HepG2 cells [45]. Moreover, both extracts contained the protocatechuic acid able to exhibit antiproliferative effects in various cell lines, including HepG2 cells [46]. However, the protocatechuic acid amount in BL was about 3 times higher than that of BS (1911.14 mg/g and 650.73 mg/g, respectively). Based on these data, it appears that the BL extract may potentially cause liver damage, and further investigations are necessary to determine its possible applications beyond the food industry. BS did not elicit any cytotoxic effects and could serve as a beneficial additive component in food and nutraceuticals. 

Yu et al. [47] explored the antioxidant properties of bamboo leaf flavonoids extracts in human HepG2 cells in which oxidative-stress was induced by oleic acid. A reduction of high ROS production and a modulation of related antioxidant defense responses carried out by bamboo flavonoids was shown, suggesting the ability of the extracts to alleviate oxidative stress in HepG2 cells. In line with these findings, Zhang et al. [48] provided evidence that bamboo leaf flavonoids suppressed hepatocellular injury and death of apoptotic cells induced by carbon tetrachloride chemical liver injury, indicating that the protective effects exhibited by this biological compound on acute liver damage are related to its strong antioxidant capacity.

In our study, we used HepG2 cells as a model system to investigate the potential antioxidant effects of BS extracts on oxidative stress induced by H_2_O_2_. It has been reported that exogenous H_2_O_2_ penetrates the cell membrane easily and generates elevated levels of free radicals which attack the mitochondrial membrane, leading to excessive ROS production in the cells [49]. In particular, the abnormal accumulation of ROS can directly induce cell damage, through the oxidation of macromolecules, such as DNA, RNA, carbohydrates, proteins and lipids, or can indirectly alter different intracellular signaling pathways implicated in the development of ageing, as well as of various chronic and degenerative diseases [50]. Therefore, antioxidants that can prevent the production of ROS are a major therapeutic means of reinforcing treatments against a wide spectrum of chronic diseases.

**Figure 3:**
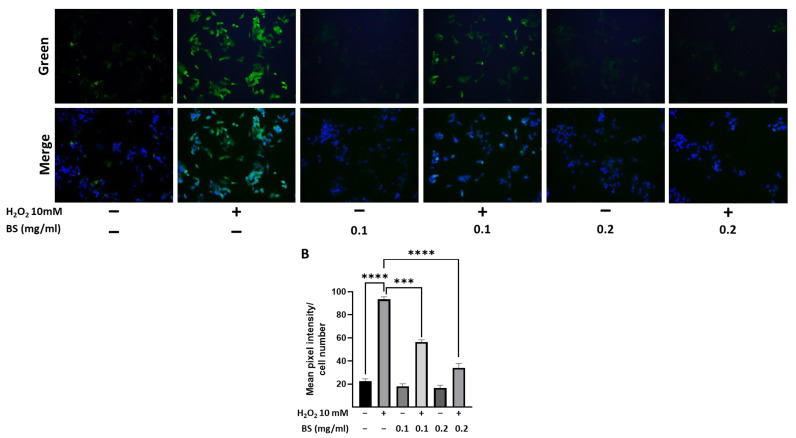
Reactive oxygen species production in HepG2 cells upon treatment with extracts from bamboo sheaths. (**A**) Representative green images (CellROX dye) of reactive oxygen species (ROS) captured by fluorescent microscopy in HepG2 cells without treatment (−) or with 0.1 and 0.2 mg/mL of bamboo sheaths (BS) for 4 h and then treated with 10 mM H_2_O_2_ for 30 min. Merged images of green and blue (DAPI (40,6-diamidino-2-phenylindole) dye for staining DNA) are shown. (**B**) ROS production is reported as mean pixel intensity (green dye) normalized to cell number (blue dye) in three independent experiments each performed in triplicate. The histograms represent means ± SEM. *** *p* < 0.001, **** *p* < 0.0001.

Our data showed that treatment with 10 mM H_2_O_2_ increased, as expected, the accumulation of ROS (*p* < 0.0001), whereas BS significantly inhibited the ROS production induced by H_2_O_2_ in a dose-dependent manner (Figure 3), suggesting that BS prevented oxidative stress damage caused by H_2_O_2_ in HepG2 cells.

### 3.4. Anti-Inflammatory Effects of Bamboo Extracts in LPS-Stimulated Human Macrophages

**Figure 4:**
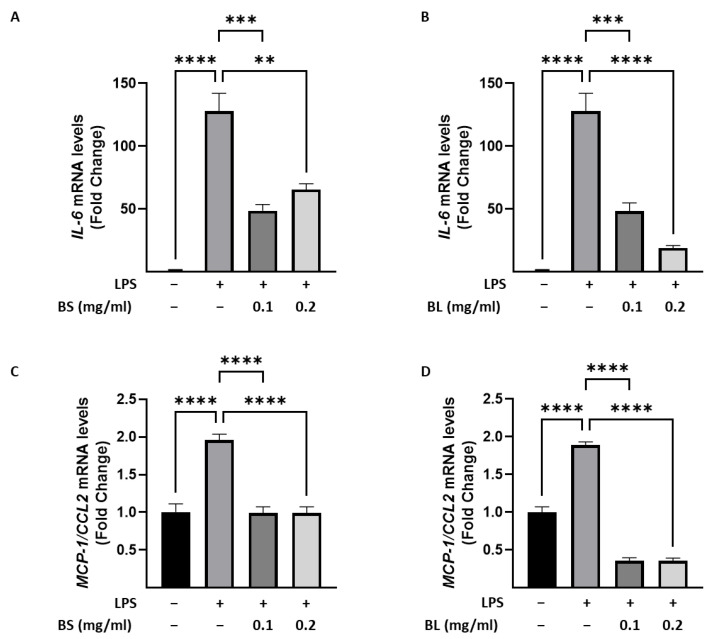
Anti-inflammatory effects of bamboo extracts in M1 macrophages. Evaluation of interleukin-6 (IL-6), Monocyte Chemoattractant Protein (MCP)-1/C-C motif chemokine ligand 2 (CCL-2) mRNA expression, by Real Time PCR assay, in macrophages untreated (−) or treated for 1 h with bamboo sheaths (BS) (**A**,**C**) and bamboo leaves (BL) (**B**,**D**) at concentrations of 0.1 and 0.2 mg/mL and then stimulated with Lipopolysaccharide (LPS) 10 ng/mL for 24 h. Data represent the means ± SEM of three different experiments each performed in duplicate. ** *p* < 0.005, *** *p* < 0.001, **** *p* < 0.0001.

Recently, it has been reported that bamboo extracts from Sasa albomarginata are able to inhibit LPS-induced inflammatory responses at nontoxic concentrations in macrophages [22]. Thus, we aimed to explore the potential anti-inflammatory activities of bamboo extracts from Phyllostacys edulis, using LPS-stimulated humanmacrophages, as an in vitro model of acute inflammation. Specifically, we evaluated by Real Time PCR the gene expression levels of pro-inflammatory cytokines in human M1 macrophages treated with 0.1 and 0.2 mg/mL of BS and BL. As shown in Figure 3, untreated macrophages, named M0 macrophages, produce low levels of Interleukin (IL)-6 (Figure 4A,B) and Monocyte Chemoattractant Protein (MCP)-1/C-C motif chemokine ligand 2 (CCL-2) (Figure 4C,D), while treatment with LPS stimulated macrophages to enhance IL-6 (Figure 4A,B) and MCP-1/CCL-2 levels (Figure 4C,D). Interstingly, BS and BL at both concentrations tested, were able to dampen the inflammation, suggesting that they have an anti-inflammatory effect in macrophages.

**Figure 5:**
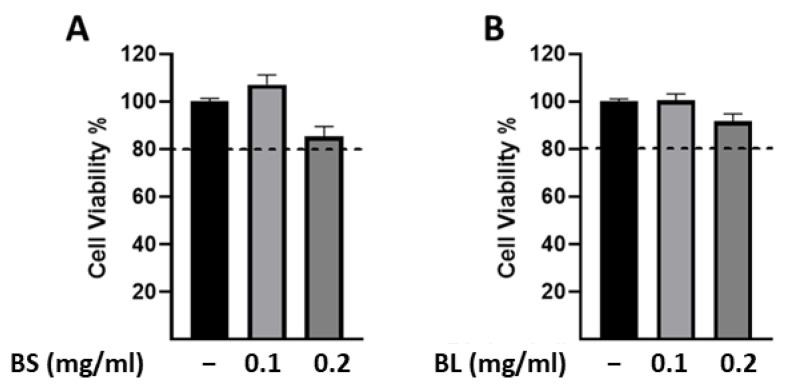
Cell viability assessed by MTT assay in human THP-1 derived macrophages. Human THP-1 derived macrophages were untreated (−) or treated with 0.1 and 0.2 mg/mL of bamboo sheaths (BS) (**A**) and bamboo leaves (BL) (**B**) for 24 h. Cell viability is expressed as percentage of control (−). The histograms represent the means ± SEMs of three different experiments, each performed in triplicate.

Importantly, to ascertain whether bamboo extracts elicit any cytotoxic effects, an MTT assay was performed in human THP-1 macrophages. The exposure of cells for 24 h to BS and BL, at concentrations of 0.1 and 0.2 mg/mL, did not affect cell viability (Figure 5), supporting the potential use of these extracts by the healthcare industry. 

Besides their application in the pharmacological industries, they can be incorporated into skincare products to help reduce skin inflammation and redness, which are often associated with conditions such as acne, rosacea and eczema. Moreover, in the food industry, BS extracts can be used as natural food additives to reduce inflammation and oxidative stress, which are known to contribute to the development of chronic diseases.

## 4. Conclusions

Our results provide insight into the antioxidant and anti-inflammatory properties of bamboo leaf and sheath processing by-products, emphasizing the potential application of this plant in food, nutraceutical, cosmetic and pharmaceutical industries. Of particular interest is that the leaf extract, characterized by a higher content of total phenols and flavonoids than the sheath processing by-products, was shown to be most active in all antioxidant assays. Moreover, both extracts impair cytokine production in a model of cellular inflammation, indicating their potential exploitation in therapeutic strategies to prevent and/or treat inflammatory diseases. Further studies are needed to investigate the mechanism of action and toxicological properties of different bamboo extracts aimed at the valorisation of plant biomass to be exploited for human health.

## Figures and Tables

**Table 1:** Protection of lipid peroxidation and Ferric Reducing Ability Power of bamboo.

Samples	β-Carotene Bleaching Test IC_50_ (µg/mL)		FRAP μM Fe (II)/g
	t = 30 min	t = 60 min	
BL	41.04 ± 4.81 ****	19.61 ± 2.58 ****	10.25 ± 2.07 ****
BS	92.48 ± 8.18 ****	79.13 ± 7.21 ****	7.71 ± 1.91 ****

BL: Bamboo leaves; BS: Bamboo sheath. Data are expressed as means ± S.D. (*n* = 3). Differences within and between groups were evaluated by One-way ANOVA followed by Dunnett’s test compared with the positive controls (propyl gallate in β-Carotene Bleaching Test (IC_50_ values of 0.09 ± 0.01 µg/mL) at both incubation times) and butylhydroxytoluene in FRAP test (FRAP value 62.27 ± 4.29 μM Fe (II)/g) (**** *p* < 0.0001).

## Data Availability

The data presented in this study are available on request from the corresponding author.

## Supplementary Materials

The following supporting information can be downloaded at: https://www.mdpi.com/article/10.3390/antiox12061239/s1, Table S1: UHPLC gradient elution. Figure S1: Chromatographic profile of bamboo sheath. Figure S2: Chromatographic profile of bamboo leaves.
