# Supplementary material for: Anti-Inflammatory and Antioxidant Effects of Leaves and Sheath from Bamboo (Phyllostacys edulis J. Houz)
Source: Antioxidants (Basel). 2023 Jun 8;12(6):1239. doi: 10.3390/antiox12061239 (PMC10294923; doi:10.3390/antiox12061239)
Supplement: Supplementary file 1 [file antioxidants-12-01239-s001.zip › antioxidants-2403275-supplementary.pdf]

Supplementary Materials

Table S1. UHPLC gradient elution.

	Time (min)	A (%)	B (%)	Flow (mL/min)
1	Initial	95	5	0.40
2	3.00	95	5	0.40
3	17.00	40	60	0.40
4	17.50	0	100	0.40
5	20.00	95	5	0.40
6	21.00	95	5	0.40

A: H_2O (pH 3.10 with acetic acid); B: CH_3CN

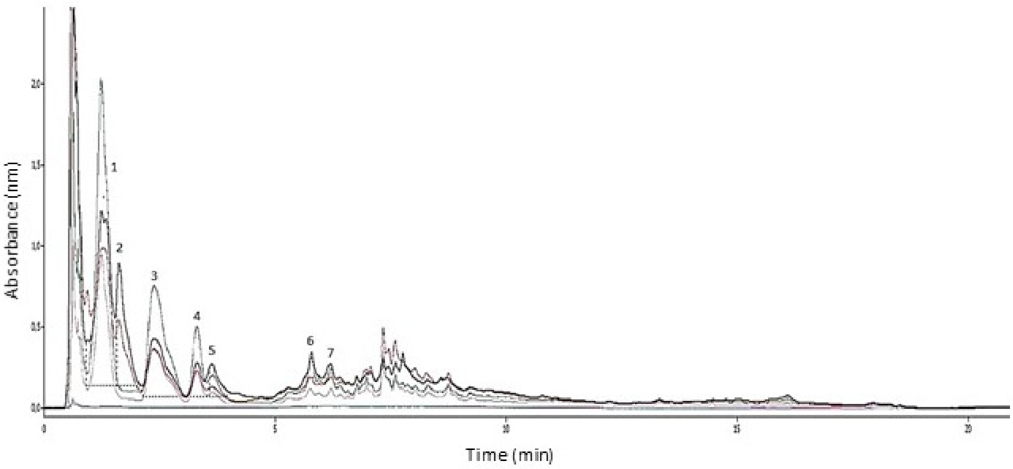

Figure S1. Chromatographic profile of bamboo sheath: 1 gallic acid; 2 protocathecuic acid; 3 chlorogenic acid; 4 caffeic acid; 5 *p*-coumaric acid; 6 ferulic acid; 7 rutin.

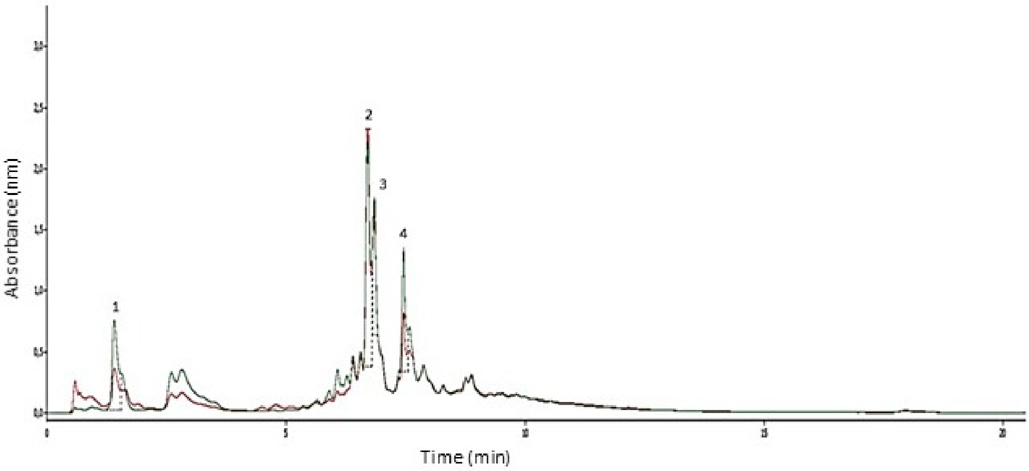

Figure S2. Chromatographic profile of bamboo leaves: 1 protocatechuic acid; 2 isoorientin; 3 orientin; 4 isovitexin.
